# How Does *Fusarium oxysporum* Sense and Respond to Nicotinaldehyde, an Inhibitor of the NAD^+^ Salvage Biosynthesis Pathway?

**DOI:** 10.3389/fmicb.2019.00329

**Published:** 2019-02-27

**Authors:** Gautam Anand, Daniel Waiger, Nuria Vital, Jacob Maman, Li Jun Ma, Shay Covo

**Affiliations:** ^1^Department of Plant Pathology and Microbiology, Robert H. Smith Faculty of Agriculture, Food and Environment, The Hebrew University of Jerusalem, Rehovot, Israel; ^2^Department of Biochemistry and Molecular Biology, University of Massachusetts Amherst, Amherst, MA, United States

**Keywords:** nicotinaldehyde, nicotinamidase, NAD^+^ biosynthesis, NAD^+^/NADH ratio, oxidoreductases, alcohol dehydrogenase, phytopathogenic fungi

## Abstract

Plant pathogenic fungi are a major threat to food security and impose a severe economic burden, thus there is a continuous need to develop new strategies to manage them. NAD^+^ is a co-factor in numerous enzymatic activities and determines the metabolic fate of the cell. Therefore, maintenance of NAD^+^ concentration is important for cellular viability. Consequently, the NAD^+^ biosynthetic pathway and redox homeostasis was suggested as a target for antifungal development. We aimed to study how *Fusarium oxysporum* senses and responds to nicotinaldehyde (NA), an inhibitor of Pnc1, a key enzyme in the salvage pathway of NAD^+^ biosynthesis. We were able to show that NA was inhibitory in high concentrations to several fungal plant pathogens, with much milder effects on tomato growth. Under low nutrient conditions NA reduced the total amounts of NAD^+^ in the fungal cell, a trend that was also observed in rich media, although without statistical significance. In low and high nutrient availability NA dramatically reduced the NAD^+^/NADH ratio. After exposure to NA, NADH levels were increased and NAD^+^ levels and the biomass were greatly reduced. Cells responded to NA by up-regulation of oxidoreductases, with hardly any up-regulation of the classic response to oxidative stress. Direct measurement of oxidative stress response showed that unlike formaldehyde and hydrogen peroxide, NA caused reductive rather than oxidative stress. Surprisingly, alcohol dehydrogenases were significantly up-regulated more than any other dehydrogenases, including aldehyde dehydrogenases. We propose that conidia of *F. oxysporum* efficiently detoxified the aldehyde group of NA by reducing NAD^+^ to NADH; the high concentrations of the latter provoked the expression of alcohol dehydrogenases that in yeast can act to reduce NADH and increase NAD^+^ amounts, respectively. Overall, the results suggest that targeting NAD^+^ biosynthesis pathway and redox homeostasis can be a potential approach to manage fungal plant pathogens. Many of the natural antifungal compounds produced by bio-control agents or even the natural biome are aldehydes, and thus the results presented here predict the possible response of *Fusarium* to wide sources of toxicity in the environment.

## Introduction

Phytopathogenic fungi have been a major threat to agriculture from ancient times. Fungal plant pathogens represent probably the most diverse group of ecologically and economically relevant threats (Doehlemann et al., 2017). *Fusarium oxysporum* (*F. oxysporum*) is a ubiquitous soil-borne pathogen that causes vascular wilt in a broad range of economically important plants. Characteristic disease symptoms include vascular browning, leaf epinasty, stunting, progressive wilting, defoliation and plant death (Agrios, 2005; Michielse and Rep, 2009). *F. oxysporum* is economically damaging to banana, threatening growth worldwide (Dita et al., 2018). Therefore, there is a constant need to develop strategies against *Fusarium* and other fungal plant pathogens. Along with resistant crops and bio-control approaches, natural and man-made chemicals are still at the front line in fighting fungal diseases. In order to develop new and effective fungicides, there is a need to study the response of fungi to different chemical stressors.

**FIGURE 1:**
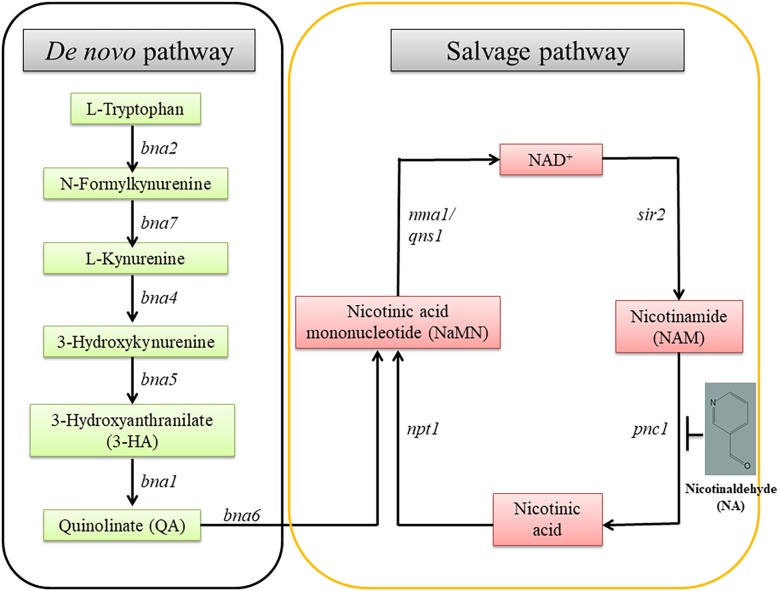
The NAD^+^ biosynthetic pathway. There are two NAD^+^ biosynthesis pathways; *de novo* and salvage. The starting point of the former is tryptophan; the latter, nicotinamide. *bna*, biosynthesis of nicotininc acid; *namn*, nicotinic acid mononucleotide; *pnc1*, pyridine nucleotide cycle 1; *sir2*, silent information regulator-2; *npt1*, nicotinate phosphoribosyl transferase 1; *nma1*, nicotinic acid mononucleotide adenyl transferase 1; *qns1*, NAD^+^ synthetase.

Pyridine nucleotides are essential metabolites for numerous redox reactions in living organisms. Nicotinamide Adenine Dinucleotide (NAD^+^) and its phosphorylated and reduced forms (NADP, NADH) are central to cellular metabolism and energy production (Sauve, 2008). Maintenance of NAD^+^ concentrations is important for cell and organism viability. NAD^+^ and NADP are important metabolites involved in cellular redox homeostasis. NAD^+^ is synthesized via two major pathways in both prokaryotic and eukaryotic systems. In one pathway, NAD^+^ is synthesized from tryptophan (the *de novo* pathway). In the other, NAD^+^ is generated by nicotinamide (NAM), nicotinic acid, nicotinamide mononucleotide (NMN) and nicotinamide riboside (NR) (the salvage pathway) (Figure 1; Handlon et al., 1994; Oppenheimer, 1994; Li and Bao, 2007; Pollak et al., 2007).

In addition to functional roles in redox regulation, recent studies show that NAD^+^ and NADP play important roles in cell signaling in animals, plants, and fungi (Hunt et al., 2004; Ziegler, 2005). NAD^+^ and NADP are the respective substrates for the production of the calcium agonists cyclic ADP-ribose (cADP-R) and Nicotinate Adenine Dinucleotide Phosphate (NaADP). Transfer of ADP-ribose units from NAD^+^ to target proteins is an important post-translational modification, catalyzed by poly (ADP-ribose) polymerases (PARPs) that functions in DNA repair. NAD^+^-dependent histone deacetylases (sirtuins) have a critical role in gene expression, DNA repair, DNA replication, lifespan, and metabolism in fungi (Bitterman et al., 2003; Howitz et al., 2003; Celic et al., 2008; Smith et al., 2008; Kawauchi et al., 2013; Muñoz-Galván et al., 2013; Simoneau et al., 2016). Sirtuins have an unusual reaction mechanism in which they consume one molecule of NAD^+^ for every lysine that is deacetylated. The acetyl group is transferred from the targeted protein to the ribose 2^′^-OH of NAD^+^, which is coupled to hydrolysis of the NAD^+^ and release of *o*-acetyl-ADP ribose and free nicotinamide (Tanny and Moazed, 2001; Zhao et al., 2004; Yuan and Marmorstein, 2012, 2013).

**Table 1:** The effect of NA exposure on the expression of genes of the NAD^+^ biosynthesis pathway and the response to oxidative stress.

Gene ID	Name	Log_2_ fold change	*p*-adjusted
**NAD biosynthesis genes**			
FOXG_01191	Npt1	0.149758	0.793213
FOXG_01512	Qns1	-0.38514	0.02431
FOXG_01665	Bna7	1.049075	7.19E-05
FOXG_01749	Bna1	0.490746	0.633839
FOXG_05660	Kynureninase 2	-0.32418	0.944792
FOXG_05832	Pnc1	0.257297	0.82261
FOXG_06167	Nma	-0.29047	0.355771
FOXG_06246	Bna6	0.742308	0.135215
FOXG_10631	Bna2	0.702356	0.924573
FOXG_11091	Pof1	0.154952	0.780328
FOXG_11506	Kynureninase 1	0.026394	0.998059
FOXG_13827	Nrk1	0.225272	0.402276
FOXG_20508	Bna4	0.674789	0.077397
**Oxidative stress response genes**			
FOXG_01738	Fe-Mn family superoxide dismutase	0.374729	0.520293
FOXG_03076	Superoxide dismutase	3.609359	8.7E-34
FOXG_04389	Fe-Mn family superoxide dismutase	-1.66711	0.125935
FOXG_13757	Fe-Mn family superoxide dismutase	-0.03881	0.998059
FOXG_17503	Cu/Zn superoxide dismutase	NA	NA
FOXG_02357	Catalase	-1.87078	0.693222
FOXG_02405	Catalase-1	0.554994	0.715415
FOXG_03915	Catalase	0.006844	0.998059
FOXG_04808	Catalase	0.083221	0.998059
FOXG_08697	Catalase	-0.18627	0.91532
FOXG_12260	Catalase-peroxidase	0.876808	0.351832
FOXG_14234	Catalase-peroxidase 2	0.50875	0.642072
FOXG_15294	Catalase	-0.82086	0.23354
FOXG_16836	Catalase	3.362096	0.333451
FOXG_17106	Catalase /peroxidase HPI	NA	NA
FOXG_17130	Catalase-peroxidase 2	-1.79091	0.970509
FOXG_17180	Catalase-peroxidase 2	2.192888	1.02E-09
FOXG_17460	Catalase-peroxidase	NA	NA
FOXG_04237	Glutathione-s-transferase	-0.90987	0.000282
**Oxidative stress response genes**			
FOXG_04328	Glutathione-s-transferase	1.032663	0.343206
FOXG_07491	Glutathione-s-transferase	0.189536	0.959633
FOXG_08382	Glutathione-s-transferase	-0.33966	0.636268
FOXG_11417	Glutathione-s-transferase	NA	NA
FOXG_11476	Glutathione-s-transferase	0.852838	0.149464
FOXG_13646	Glutathione-s-transferase	-0.79152	0.31319
FOXG_13780	Glutathione-s-transferase	1.054219	0.330752
FOXG_14789	Glutathione-s-transferase	-0.04109	0.998059
FOXG_16651	Glutathione-s-transferase	1.690639	0.012985
FOXG_16708	Glutathione-s-transferase	NA	NA
FOXG_16775	Glutathione-s-transferase	NA	NA
FOXG_19079	Glutathione-s-transferase	NA	NA
FOXG_07868	Glutathione peroxidase	0.746943	0.454915
FOXG_13439	Heme-binding peroxidase	1.598157	0.00092


The role of NAD^+^ in the activity of sirtuins and PARP provides a link between metabolism, the cellular redox state, chromatin regulation and post-translational modifications of non-histone proteins. The byproduct of sirtuins and other NAD^+^ consumers is nicotinamide, which is recycled to NAD^+^ via the NAD^+^ salvage biosynthesis pathway. A key enzyme in this reaction is Pnc1, a nicotinamidase which converts nicotinamide to niacin. Pnc1 was shown to be involved in longevity and redox control in yeast (Anderson et al., 2003; Radzinski et al., 2018). Thorough structural and biochemical investigation led to the identification of nicotinaldehyde (NA) as an inhibitor of Pnc1 at micromolar levels (Figure 1; Smith et al., 2012). NA binds to catalytic cysteine residues of nicotinamidases through thiohemiacetal linkage. In addition to covalent linkage to cysteine, NA also binds to the zinc ion in the active site through its nicotinic ring. Binding of NA to Pnc1 and the attack of the cysteine at the Pnc1 active site on the aldehyde resembles very much the binding and catalytic activity of Pnc1 on its natural substrate. Yet, this catalysis is futile. Therefore, ketones and aldehyde containing nicotinamide analogs can be potent inhibitors of Pnc1 (French et al., 2010a,b; Smith et al., 2012). It was suggested that NA may serve as a fungicide. This notion is somewhat supported by the fact that nicotinamide by itself is toxic to *Candida albicans* and that inhibition of Pnc1 is expected to increase intracellular amounts of nicotinamide (McClure et al., 2008; Wurtele et al., 2010). Pnc1 supports increase in cellular NAD(H) levels in response to internal or external oxidative stress (Anderson et al., 2003).

Here by using NA, we have tried to inhibit the NAD^+^ biosynthetic pathway in the plant pathogen *F. oxysporum* with the aim to inhibit its growth. Nothing is known about the role of the NAD^+^ cycle in the biology and phytopathology of plant pathogenic fungi and in filamentous fungi altogether. In addition to its Pnc1 inhibitory properties, the aldehyde group of NA is also expected to have a toxic effect. Very little is known about the response of fungal plant pathogens to aldehydes despite their environmental and metabolic relevance and the fact that the *F. oxysporum* genome encodes for more than twenty aldehyde dehydrogenases (Singh et al., 2013; Shreaz et al., 2016; Abdul et al., 2018).

## Materials and Methods

### Culture Growth and Treatment

Unless stated otherwise in this work we used *F. oxysporum* f.sp *lycopersici* 4287 (*F. oxysporum* for short). *F. oxysporum* cultures were grown in KNO_3_ media [1.36 g yeast nitrogen base, 24 g sucrose, 100 mM KNO_3_ in 800 ml distilled water (DDW)] for 5 days. The cultures were filtered to obtain conidia that were washed in DDW. For majority of experiments, 2.5 × 10^7^ conidia/ml were grown in potato dextrose broth (PDB, BD, Sparks, NV, United States) or DDW for different time intervals with or without addition of NA (Sigma).

### Effect of NA on Conidial Germination and Hyphal Growth

The 2.5 × 10^7^ conidia/ml of *F. oxysporum* were grown in PDB or DDW in 50 ml culture flasks. Up to 10 mM of NA were added to both PDB and DDW cultures, which were then allowed to grow for 14 and 42 h, respectively. Germinated and ungerminated conidia were counted using a Neubauer chamber. To study the effect of NA on hyphal growth, mycelial agar plugs were placed on potato dextrose agar (PDA) plates with NA concentrations ranging 0–10 mM. Plates were incubated at 28°C for 5 days. Then, the plates were scanned and the radius of the fungal colony was measured.

### RNA Extraction

*Fusarium oxysporum* conidia were inoculated in PDB, after 5 h NA was added to a final concentration of 10 mM followed by a further incubation for 3 h. As a control, conidia were inoculated in PDB and grown for 8 h. Cells were disrupted using Minilys bead beater for 30 s at medium speed. RNA was purified using the RNeasy Plant Mini Kit of QIAGEN (Hilden, Germany) and was treated on-column with RQ1 RNase-free DNase (Promega Corp., United Sates) to remove additional residues of genomic DNA. RNA quality was measured by a tape station machine and kit (Santa Clara, CA, United States). RNA was submitted to the Crown Institute of Genomics of the G-INCPM (Weizmann Institute of Science, Israel) and RNA sequencing libraries were made using the TrueSeq Kit according to manufacturer instructions (Illumina, San Diego, CA, United States). Next, the libraries were sequenced on one lane of a HiSeq 2500 machine.

### Sequencing and Analysis

Sequence reads were aligned to the *F. oxysporum* 4287 (FO2) genome (downloaded on 26/Aug/2015 from the Broad’s Institute Fusarium comparative genome project) using TopHat (v2.0.10) (Kim et al., 2013). HTSeq-count (version 0.6.1p1) (Anders et al., 2015) was used to count reads on genes. Differential expression analysis was performed using DESeq2 (1.6.3) (Love et al., 2014) with betaPrior set to False. Upregulated genes following NA exposures were subjected to STRING analysis (version 10.5) with a confidence of 0.7 (Szklarczyk et al., 2017).

### Measurement of Oxidative Stress

To evaluate *F. oxysporum* oxidative stress generated by NA (10 mM) and formaldehyde (10 mM), reactive oxygen species (ROS) generation was measured using cell permeant fluorogenic dye 2^′^, 7^′^-dichlorofluorescin diacetate (DCFDA) (Abcam, United Kingdom). Briefly, the DCFDA dye loses its fluorescence upon enzymatic processing within the cell. Exposure to cellular ROS oxidizes the molecule into 2^′^, 7^′^-dichlorofluorescein which is highly fluorescent. 1 × 10^6^ conidia/ml were grown in PDB for 2 h, conidia were then washed by centrifugation in 1× Buffer provided with the kit. DCFDA (40 μM) and NA or formaldehyde were added. As a positive control, conidia were treated with Tert-Butyl Hydroperoxide (TBHP) at final concentration of 0.1 mM. Conidia were incubated for 30 min in complete darkness at 37°C. Fluorescence was measured (Ex/Em = 485/535 nm) using Spark 10 M plate reader of Tecan (Männedorf, Switzerland).

### Measurement of Nicotinamidase Activity

To study the effect of NA on nicotinamidase activity, whole cell extracts from *F. oxysporum* were prepared using lyticase enzyme (Sigma) in citrate-phosphate buffer (pH 5.5). The mixture was incubated at 37°C for 45 min followed by bead-beating for 60 s. The extract was then centrifuged at 10,000 rpm for 10 min at 4°C. The obtained supernatant was used to measure the enzymatic activity. To measure nicotinamidase activity indirectly, an ammonia release assay was used (Hunt et al., 2007). When nicotinamide is hydrolyzed by nicotinamidase, ammonia is released. Ammonia is used to convert α-ketoglutarate to glutamate; NADH is oxidized to NAD^+^ in the process (Illustrated in Figure 6A). The decrease in NADH levels that is corresponding to the oxidation of NADH produced by glutamate dehydrogenase is measured by reduction in absorbance at 360 nm (Smith et al., 2009). Absorbance was measured at 360 nm instead of 340 nm to reduce the interference of NA and due to the amount of NADH used to saturate glutamate dehydrogenase. The standard reaction medium (200 μl) for the above assay contained 250 μM NADH, two units glutamate dehydrogenase, 0.5 mM nicotinamide, 1 mM α-ketoglutarate and 10 μl of conidia extract in 50 mM tris-buffer (pH 8.0). One unit of activity is defined as the amount of enzyme consuming 1 mmol of NADH in 1 min at pH 8.0 and 37°C. As a control, an ammonia release assay was performed using conidia extract but without NAM and/or α-ketoglutarate in order to determine the presence of any other NADH-consuming enzymes – very low nicotinamidase activity was detected under these conditions. To measure the effect of NA on enzyme activity, NA at a final concentration of 10 mM was added to the reaction mixture (containing NAM and α-ketoglutarate).

### NAD^+^/NADH Extraction and Quantification

For NAD^+^/NADH extraction from *F. oxysporum*, 2.5 × 10^7^ conidia/ml were grown in PDB or water for a given time, and then NA was added to a final concentration of 10 mM for another 3 h. As a control, instead of adding NA, conidia were further grown for another 3 h. NAD^+^/NADH quantification from conidia was also performed in response to 10 mM formaldehyde and 0.1 mM hydrogen peroxide (H_2_O_2_). For H_2_O_2_ treatment, conidia were grown for 2 h in PDB followed by the addition of H_2_O_2_ for another 30 min. NAD^+^/NADH from *F. oxysporum* were extracted using a buffer that contained 50 mM Tris (pH 7.9), 100 mM ammonium acetate, 10 mM DTT and 5% glycerol. Cells were disrupted by two rounds of bead-beating for 30 s followed by incubation on ice for 2 min. The extract was then incubated on ice for 15 min. During incubation cells were vortexed for 5 s every 5 min to promote the extraction. The supernatant obtained after centrifugation (10,000 rpm, 10 min, 4°C) was used for NAD^+^/NADH quantification.

Total NAD (NADH + NAD^+^) in the supernatant was detected using BioVision NAD^+^/NADH Quantification Colorimetric Kit (BioVision, CA, United States) with some modifications. To measure NADH only, 0.1 M NaOH was added and the cell extract was heated at 60°C for 30 min followed by neutralization with 0.1 M HCl. Alkaline treatment and heating degraded NAD^+^ and the NADH left in the extract was quantified according to the manufacturer instructions. Total protein in the extract was measured by Bradford method with bovine serum albumin as standard (Bradford, 1976).

### Combined Effect of NA and Boscalid on *F. oxysporum* Growth

Effect of NA and boscalid (Sigma) on growth of *F. oxysporum* was studied by measuring the radius of the fungal colony after dropping 1,000 conidia at the center of petri dishes (90 mm filled with 20 ml of agar media) containing PDA with or without 1 mM NA and/or 10 μg/ml boscalid. The plates were incubated for 5 days at 28°C and then scanned.

### Effect of NA on Growth of Different Phytopathogenic Ascomycetes and Tomato Plants

Effect of NA on growth of *F. oxysporum* f.sp *melonis*, *F. verticilliodes*, *Botrytis cinerea*, and *Penicillium expansum* was studied by measuring the radius of the fungal colonies. Petri dishes (90 mm filled with 20 ml of agar media) containing PDA with or without 10 mM NA were used. 10,000 fungal conidia were placed in each half of a PDA plate. The plates were incubated at 25°C for 5 days and then scanned.

To study the effect of NA on tomato plants, seeds of Rehovot 13 tomatoes were germinated in soil for several days in the greenhouse (Katan, 1971). Tomato seedlings were further grown in the greenhouse for 3 days in water with standard fertilizer in a hydroponics setup. Next, NA and nicotinamide were added to their final concentration (10 and 100 mM, respectively). The tomato plants were grown for another 8 days.

### Inoculation of Disconnected Leaves With *B. cinerea* Conidia

To measure the efficiency of NA as a protectant seven Rehovot 13 tomato leaves were briefly soaked in 10 mM NA or water. Next, the leaves were inoculated with 5000 conidia of *B. cinerea* resuspended in 2% glucose solution. The inoculated leaves were incubated in petri dishes containing water agar in a moist chamber at 25°C for 4 days. The lesion size was measured using ImageJ software package. To measure the efficiency of NA as a systemic agent, seven leaves from plants that were grown in hydroponic setting (see above) without treatment and seven that were grown in 10 mM NA were inoculated with *B. cinerea* as described above.

### Statistical Analysis

For statistical analysis, the Student’s *t*-test and two-tailed Chi-square test were implemented in SPSS 15.0 software (IBM Corporation). *P* ≤ 0.05 was chosen as the cut-off point for statistical significance.

## Results

### Nicotinaldehyde Shows *in vitro* Antifungal Activity

**FIGURE 2:**
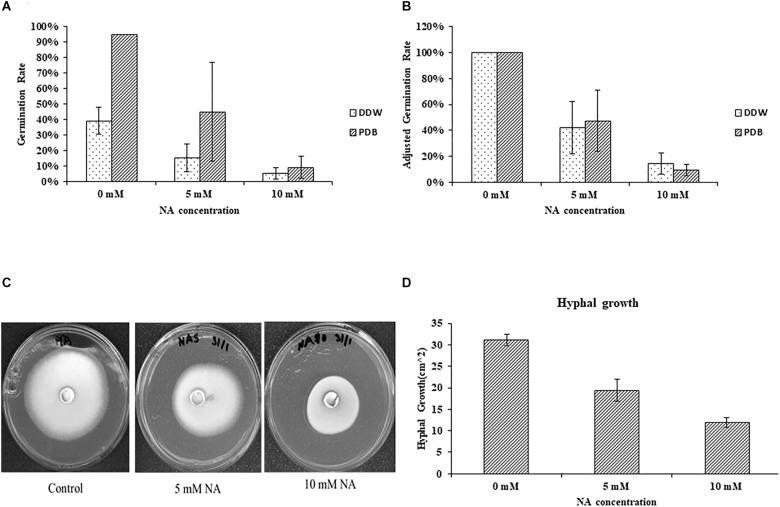
Nicotinaldehyde (NA) has a greater inhibitory effect on germination of *F. oxysporum* than on hyphal growth. **(A,B)** Effect of different concentrations of NA (0–10 mM) on conidial germination in PDB and double distilled water (DDW). Conidia were inoculated in PDB and DDW for 14 and 42 h, respectively. Germination was determined as described in Materials and Methods section. Values are mean ± SD and statistically significant with respect to control (*p* < 0.05). Vertical lines represent standard error bar. **(C)** Effect of different concentrations of NA on hyphal growth. Plates were incubated at 28°C for 5 days and then scanned. **(D)** Graphical representation of the inhibitory effect of NA on hyphal growth. Values are mean ± SD and statistically significant (*p* < 0.05). Vertical lines represent standard error bars.

Nicotinaldehyde was previously shown to effectively inhibit Pnc1 in yeast, and NA was suggested as a potential anti-fungal compound (Smith et al., 2012). To the best of our knowledge, the inhibitory effect of NA was never studied in filamentous fungi. To this end, we first studied the effect of NA on germination of *F. oxysporum* conidia. Since NA should inhibit the salvage pathway of NAD biosynthesis, we used two conditions: (1) DDW, where no nutrients are available and NAD^+^ biosynthesis should rely only on components stored in the conidia, and (2) PDB, where nutrients are available and NAD^+^ can be synthesized from external sources. The effect of NA on germination of *F. oxysporum* was studied in DDW over 42 h. There was a significant reduction in germination with the increase in NA concentrations (Figure 2A,B). At 10 mM, 85% reduction in germination was observed compared to the control. NA effectively inhibited the germination of *F. oxysporum* conidia in PDB medium over 14 h of incubation where the overall germination rate was twice as high as the rate in water (Figure 2A,B). In PDB, the germination of *F. oxysporum* was reduced to 45 and 10% at 5 and 10 mM NA concentrations, respectively (Figure 2A,B). Taken together, NA inhibited fungal development, albeit at high concentrations under rich and nutrient-free conditions. It should be noted that at the starting point of the germination experiments the conidia suspension included about 4% of carry-over germilings. These observations were obtained when either 40 μm pore cell strainer or 10 μm pore miracloth were used. Next, we studied the effect of NA on mycelial growth. The inhibitory effect of NA on mycelia was milder, with only 33 and 66% inhibition in the presence of 5 and 10 mM NA, respectively (Figure 2C,D). At this point we do not understand the reason for the differences between inhibition of germination and mycelia development by NA. The difference is probably not due to the different cultivation conditions on solid and in liquid media. When 10,000 conidia of *F. oxysporum* f.sp *lycopersici* were inoculated at the center of a PDA plate containing 10 mM NA no growth was observed. In addition, inoculation of an agar plug covered with *F. oxysporum* mycelium in PDB or PDB containing 10 mM NA resulted only in mild inhibition (Supplementary Figure S1).

### Transcriptomic Response of *F. oxysporum* to NA

**FIGURE 3:**
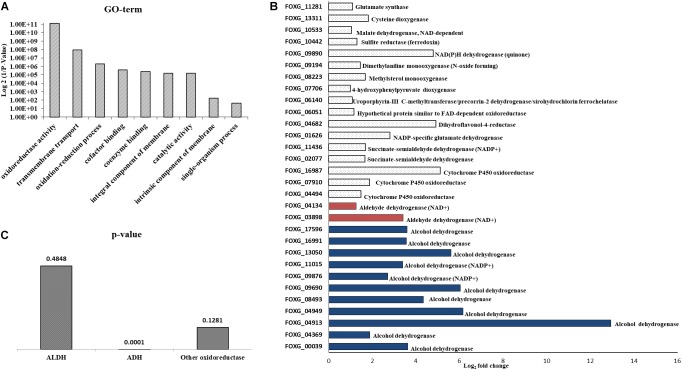
RNA sequencing of conidia exposed to NA revealed upregulation of oxidoreductases. **(A)** GO-term analysis of differentially upregulated genes following NA exposure. **(B)** Alcohol dehydrogenases are the major oxidoreductases to be upregulated as compared to other oxidoreductases. Upregulation of all genes presented is statistically significant with *p* < 0.05 as predicted by Chi-square *t*-test. **(C)** *p*-value graph of oxidoreductases among all the upregulated genes (annotated and hypothetical). Alcohol dehydrogenases are most significantly upregulated when conidia were treated with 10 mM NA. ALDH, aldehyde dehydrogenases; ADH, alcohol dehydrogenases.

The NA doses required to inhibit fungal development were high; it was unclear how much NA got into the cell and how much was detoxified. If high concentrations of NA penetrate the cell, the associated fungal growth may not be related to its Pnc1 inhibitory effect. To better understand the mode of toxicity of NA and the fungal response to NA stress, we performed an RNA sequencing experiment. Conidia were inoculated in PDB for 5 h to break dormancy, then either treated with 10 mM NA or not treated at all. RNA was harvested and sequenced using Illumina sequencing as described under Materials and Methods. A total of 718 genes were significantly upregulated and 641 were significantly downregulated (Supplementary Tables S1, S2). Genes that are related to oxidation-reduction enzymatic activities (oxidoreductases) were induced in the presence of NA, as revealed by GO term analysis (Figure 3A). Other enriched GO terms were transmembrane transport and co-factor binding (Figure 3A). Up-regulation of oxidoreductases is in agreement with inhibition of the NAD^+^ biosynthesis pathway since NAD^+^ is the major redox determinant in the cell and since most oxidoreductases use NAD^+^ or NADH as a cofactor. However, the aldehyde group of NA could also provoke oxidoreductase expression. Many dehydrogenases, such as aldehyde dehydrogenases (FOXG_03898, FOXG_04134), alcohol dehydrogenases (FOXG_04369, FOXG_04913, FOXG_04949, FOXG_08493, FOXG_09690, FOXG_09876, FOXG_13050, FOXG_16991, FOXG_17596), and succinate dehydrogenase (FOXG_02077, FOXG_11436), were induced (Figure 3B). Surprisingly, among the annotated genes, the induction of alcohol dehydrogenases was statistically more significant (*p* = 0.0001) than aldehyde dehydrogenases (*p* = 0.045). In fact, if all of the upregulated genes (annotated and hypothetical) were considered, alcohol dehydrogenase was the only group of dehydrogenases that was significantly enriched (Figure 3C). In addition, cytochrome P450 oxidoreductases (FOXG_04494, FOXG_07910, FOXG_16987), which likely contribute to other redox pathways through their ability to reduce cytochrome b5, were upregulated in the presence of NA. Only one gene in the NAD^+^ pathway as described in yeast was upregulated in response to NA (*bna7*, FOXG_01665, Table 1). Many genes encoding proteins acting against oxidative stress, such as glutathione-s-transferase, catalases and superoxide dismutase, were not induced (Table 1). Among different catalases, only one (FOXG_17180) was found to be upregulated. Also, only a single superoxide dismutase (FOXG_03076) and glutathione-s-transferase (FOXG_16651) were induced (Table 1). Thus, the classic oxidative stress response was not induced under these conditions. In addition, we could not observe a classic response to growth arrest that is usually manifested by reduction in the expression of ribosomal proteins (Zhao et al., 2003).

**FIGURE 4:**
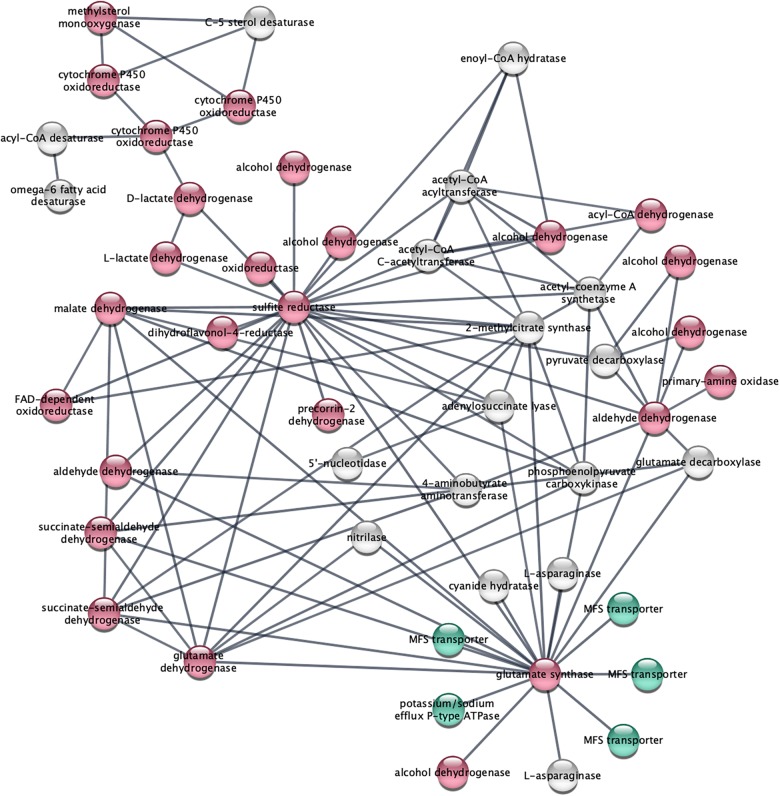
STRING analysis of upregulated genes depicts a network of dehydrogenase genes, with sulfite reductase (FOXG_10442) and glutamate synthase (FOXG_11281) in the center. Upregulated genes following NA exposures were analyzed using the STRING platform as described in Materials and Method. Nodes colored in red are oxidoreductases; nodes colored in green are transport proteins. The gene IDs that match the gene descriptions presented in the figure are found in Supplementary Table S3.

STRING analysis of functional interactions identified a network of proteins among the upregulated genes (Figure 4 and Supplementary Table S3). Sulfite reductase (FOXG_10442) and glutamate synthase (FOXG_11281) were identified in the center of this network that included dehydrogenases and other oxidoreductases.

### NA Does Not Induce Oxidative Stress

**FIGURE 5:**
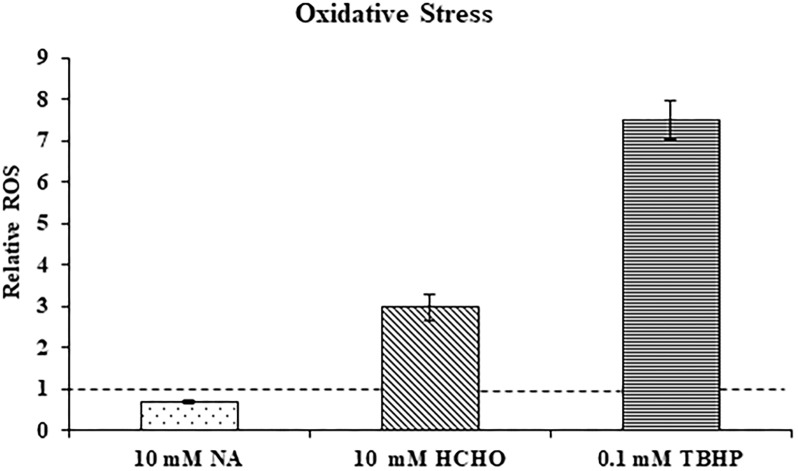
NA does not induce oxidative stress. Relative levels of reactive oxygen species (ROS) in *F. oxysporum* conidia growing in PDB in response to 10 mM NA, 10 mM formaldehyde (HCHO), and 0.1 mM TBHP as measured by the DCFDA kit (see Section “Materials and Methods”). The dotted line represents the ROS status of untreated control. Values are mean ± SD and statistically significant with respect to control (*p* < 0.05). Vertical lines represent standard error bars.

Transcriptome study revealed that genes that were part of the oxidative stress response were not significantly induced in *F. oxysporum* in response to NA. This might suggest that NA did not induce oxidative stress. Next, ROS generation in response to NA was directly measured using the DCFDA method (see Materials and Methods). ROS generation was also measured when cells were exposed to formaldehyde (HCHO) and Tert-Butyl Hydroperoxide (TBHP) as a positive control for oxidative stress. It was found that 30 min exposure to formaldehyde and TBHP generated approximately 3.0 and 7.5 times more ROS in *F. oxysporum* than in the untreated conidia. Surprisingly, after NA treatment ROS levels decreased significantly by 29.4% compared with the untreated conidia (Figure 5). In conclusion, our data suggested that NA did not induce oxidative stress in *F. oxysporum*; rather, it generated reductive stress.

### NA Disrupts NAD^+^ Biosynthesis in *F. oxysporum* and Disturbs the Redox Ratio (NAD^+^/NADH)

**FIGURE 6:**
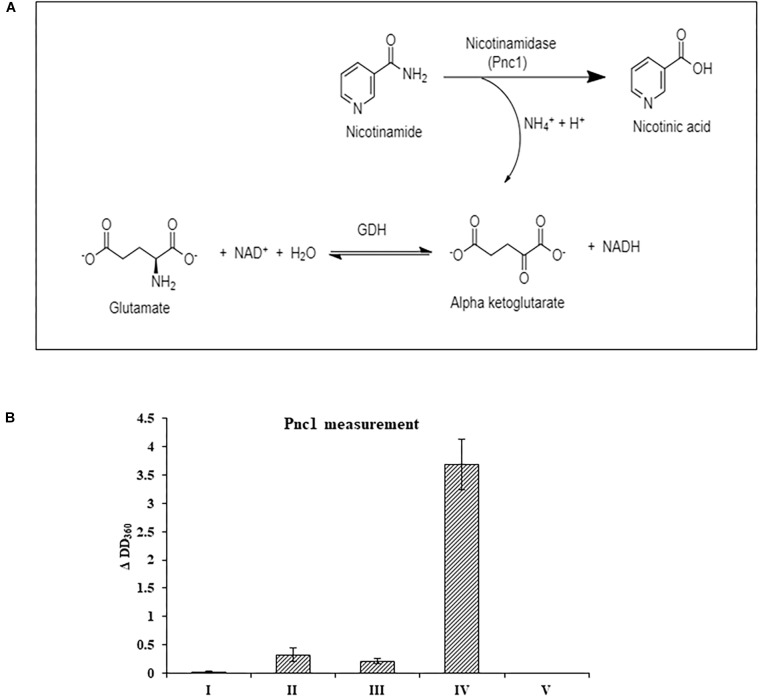
The effect of NA on nicotinamidase activity in extracts of *F. oxysporum.* **(A)** An assay to measure nicotinamidase activity that is attributed to Pnc1. GDH, glutamate dehydrogenase **(B)** Nicotinamidase activity is completely inhibited by 10 mM NA. Nicotinamidase activity was measured as described in Materials and Methods. Values are mean ± SD and statistically significant with respect to control (*p* < 0.05). Vertical lines represent standard error bars. I, without fungal extract; II, without nicotinamide and α-ketoglutarate; III, without nicotinamide; IV, with NAM, α-ketoglutarate and fungal extract; V, as in IV, but with 10 mM NA.

At the RNA level, neither the NAD^+^ *de novo* nor salvage biosynthesis pathways were induced by NA exposure. Therefore, the effect of NA on nicotinamidase activity (catalyzed by Pnc1) was studied. The assay performed was an indirect measurement of a nicotinamidase activity. The assay measures indirectly the decrease of NADH levels (see Section “Materials and Methods” and Figure 6A). We were able to observe significant activity in *Fusarium* extract that was dependent both on nicotinamide and α-ketoglutarate, indicating that the NADH concentration changes that were measured were the result of nicotinamide hydrolysis (Figure 6B). 10 mM NA completely abolished nicotinamide-dependent reduction of NADH concentrations in agreement with inhibition of nicotinamidase activity (Figure 6B).

**FIGURE 7:**
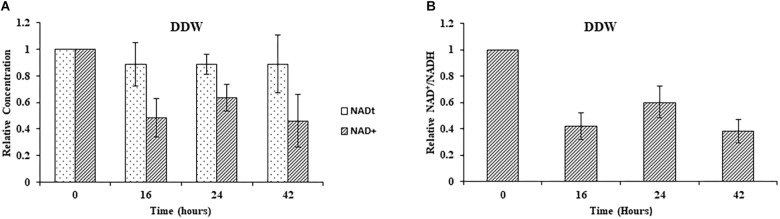
Changes in total NAD levels and NAD^+^ oxidation status during germination of conidia under nutrient-free conditions. **(A)** Relative concentrations of total NAD (NADH + NAD^+^) and NAD^+^ in *F. oxysporum* conidia growing in DDW at different time intervals. **(B)** Redox ratio of *F. oxysporum* conidia growing in DDW for different time intervals. NAD was extracted and measured as described in Materials and Methods. Vertical bars represent relative values with respect to initial concentration at 0 h. Values are mean ± SD. Vertical lines represent standard error bars.

**FIGURE 8:**
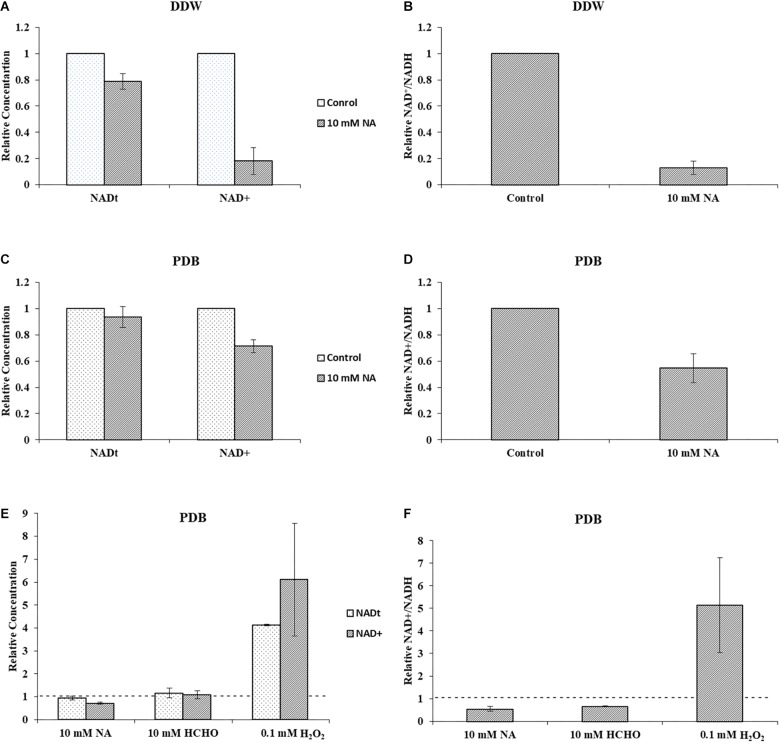
NA inhibits NAD^+^ biosynthesis and disturbs NAD^+^/NADH ratio in *F. oxysporum.* **(A)** The effect of 10 mM NA on total NAD and NAD^+^ concentrations in DDW is presented. **(B)** The effect of 10 mM NA on NAD^+^/NADH ratio in DDW is presented. **(C)** The effect of 10 mM NA on total NAD and NAD^+^ concentrations in PDB is presented. **(D)** The effect of 10 mM NA on NAD^+^/NADH ratio in PDB is presented. **(E)** Effect of 10 mM NA, 10 mM formaldehyde (HCHO) and 0.1 mM H_2_O_2_ on total NAD and NAD^+^ concentration of conidia grown in PDB. **(F)** Effect of 10 mM NA, 10 mM HCHO, and 0.1 mM H_2_O_2_ on NAD^+^/NADH ratio. Conidia were grown in DDW and PDB for 24 and 5 h, respectively, followed by 3 h of NA exposure. To study the effect of HCHO, conidia were grown in PDB for 5 h, followed by 3 h of HCHO exposure. To study the effect of H_2_O_2_, conidia were grown in PDB for 2 h, followed by 30 min of H_2_O_2_ exposure. NAD was extracted and measured as described in Materials and Methods. The vertical bars represent relative values with control as reference. Dotted lines represent untreated controls. Values are mean ± SD. Vertical lines represent standard error bars.

Next, we studied the effect of NA on NAD^+^ amounts and redox ratio. First, these parameters were measured in *F. oxysporum* conidia germinated in water at different time intervals (0, 16, 24, and 42 h). Interestingly, the total NAD^+^ (NADH + NAD^+^) did not change significantly for 42 h (Figure 7A). The NAD^+^ levels and therefore NAD^+^/NADH ratio decreased at 16 h and remained constant until 42 h (Figure 7A,B). The effect of NA on NAD^+^ biosynthesis in *F. oxysporum* was studied in DDW (Figure 8A,B). It was found that at 10 mM NA there was a significant reduction in the NAD^+^ levels. A remarkable decrease of 82% was observed in NA-treated cells with respect to control (Figure 8A). Total NAD significantly decreased by more than 21% (Figure 8A). There was also a change in the redox ratio (NAD^+^/NADH) of the cells of approximately 80% in the NA-treated cells with respect to the control (Figure 8B). The effect of NA on NAD^+^ biosynthesis was also studied in PDB. A trend of reduction in total NAD level was observed, with a decrease of more than 28% in NAD^+^ concentration, although lacking statistical significance (Figure 8C). Also, the redox ratio decreased significantly by more than 45% (Figure 8D). 10 mM formaldehyde on the other hand increased the total NAD and NAD^+^ levels by 16.5 and 7.4% (lacking statistical significance though) (Figure 8E). The NAD^+^/NADH ratio decreased significantly by more than 34% following formaldehyde exposure (Figure 8F). In contrast to NA, 0.1 mM H_2_O_2_ caused a tremendous increase in total NAD, NAD^+^ levels and NAD^+^/NADH ratio (Figure 8E,F). This increase was indicative of oxidative stress. To sum up, the effect of NA on NAD^+^/NADH was similar to formaldehyde but very different from H_2_O_2_. However, while NA caused a reduction in NAD^+^ concentrations, formaldehyde caused an increase.

### Inverse Correlation Between NADH Level and Biomass

**FIGURE 9:**
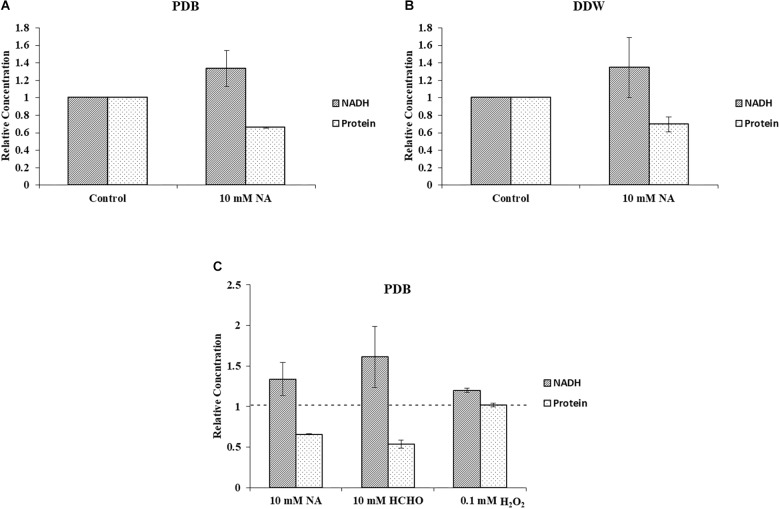
NA increases level of NADH and decreases *F. oxysporum* biomass. **(A)** Inverse correlation between NADH and total protein in cultures of *F. oxysporum* grown in PDB with 10 mM NA. **(B)** Inverse correlation between NADH and total protein in cultures of *F. oxysporum* grown in DDW with 10 mM NA. **(C)** Relation of NADH levels and cellular protein content of *F. oxysporum* grown in PDB with 10 mM NA, 10 mM HCHO, or 0.1 mM H_2_O_2_. Vertical bars represent relative concentrations of NADH and protein with respect to control as reference. Dotted line represents untreated control. Values are mean ± SD. Vertical lines represent standard error bars.

Quantification of cellular NADH and protein concentrations from *F. oxysporum* grown under 10 mM NA revealed a correlation between NADH levels and biomass (Figure 9A,B). This result was consistent for cultures that were grown in PDB or DDW. When conidia were gown in PDB with 10 mM NA, NADH levels increased by 33.6% with respect to the control, whereas total cellular protein decreased by 34.3%. In DDW cultures, NADH levels increased by 34.4% and the total protein concentration decreased by 30.7% with respect to the control. Similar trend was observed when conidia were treated with 10 mM formaldehyde. NADH levels were increased by more than 61% whereas the protein concentration was decreased by 46.5% (Figure 9C). We found a 20.0% increase in NADH levels when conidia were treated with 0.1 mM H_2_O_2_ without an effect on protein concentrations (Figure 9C). The absence of any effect on protein concentrations following H_2_O_2_ treatment could stem from the huge increase in NAD^+^ levels (more than 600%) which was also responsible for the increase in the NAD^+^/NADH ratio (Figure 8E,F). These results are further discussed below. Briefly, we think that the role of upregulated alcohol dehydrogenases following NA exposure (Figure 3) is to reduce the NADH levels.

### Additive Effect of NA and Boscalid

**FIGURE 10:**
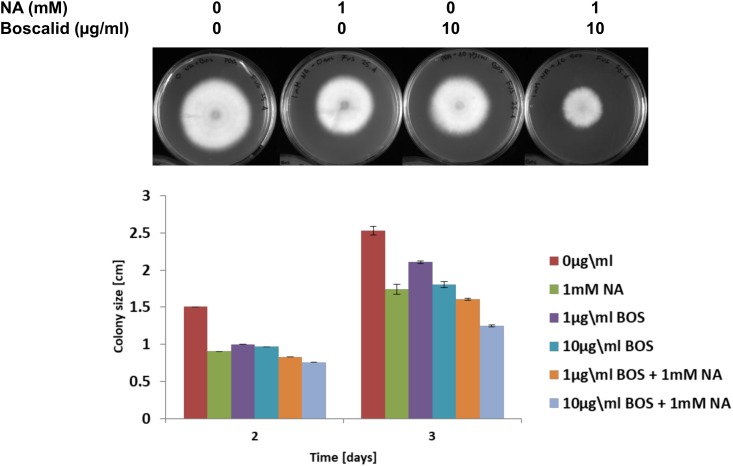
The effect of NA and boscalid on *F. oxysporum* growth. 1000 conidia were dropped at the center of PDA plates containing the indicated concentrations of NA and boscalid. The surface area of the colony was calculated from its radius.

We present here an inhibitory effect of NA on fungal growth at high concentrations. Since our transcriptomic data together with NAD^+^/NADH and ROS measurements suggested that NA interfered with cellular redox and metabolism, we thought that combination with a fungicide that interferes with metabolism in a different way may allow reducing the dosage of NA. Indeed, the combination of low-dose NA with the succinate dehydrogenase inhibitor boscalid resulted in additive inhibition. The surface size of colonies developed from conidia inoculated on PDA plates containing boscalid and NA was smaller than the surface size of the colonies that were developed from conidia inoculated on PDA plates containing each of these chemicals alone (Figure 10).

### NA Inhibits Growth of Phytopathogenic Ascomycetes More Than Tomato Plants

**FIGURE 11:**
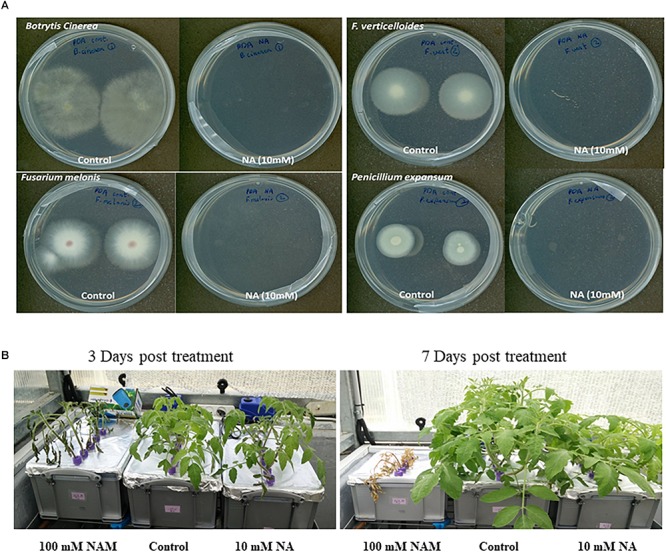
The effect of NA on fungal plant pathogens and tomato plants. **(A)** Conidia from different plant pathogenic ascomycetes were dropped at the center of PDA plates with or without 10 mM NA. **(B)** Tomato seedlings were grown for 3 days in water with standard fertilizer in a hydroponic setup. NA and nicotinamide were then added to their final concentration (10 and 100 mM, respectively). The tomato plants were grown for another 8 days.

The NA effect on growth of different plant pathogenic ascomycetes, namely *B. cinerea*, *F. oxysporum* f.sp *melonis*, *F. verticillioides*, and *P. expansum*, was studied. Conidia of the above fungi were dropped on plates with or without 10 mM NA. The plates were further incubated for 5 days. The colony formation of conidia inoculated on NA plates was completely inhibited (Figure 11A). There was no visible growth of any of these fungi on NA plates. This result shows that NA is very effective in suppressing the growth of phytopathogenic ascomycetes *in vitro*. We also studied the effect of NA on tomato growth in a hydroponic setting. Tomato seedlings were grown for 1 week in water, 100 mM nicotinamide, or 10 mM NA. 100 mM nicotinamide had a similar effect on fungal growth as 10 mM NA (data not shown). While 100 mM nicotinamide was completely toxic to plants, 10 mM NA only inhibited growth (Figure 11B). The effect of NA on plant growth was not seen 3 days post treatment. Therefore, finding ways to reduce NA amounts without reducing its activity may be a basis for future fungicide development.

### NA Suppresses Disease Symptoms Caused by *B. cinerea* in a Disconnected Leaf Assay

**FIGURE 12:**
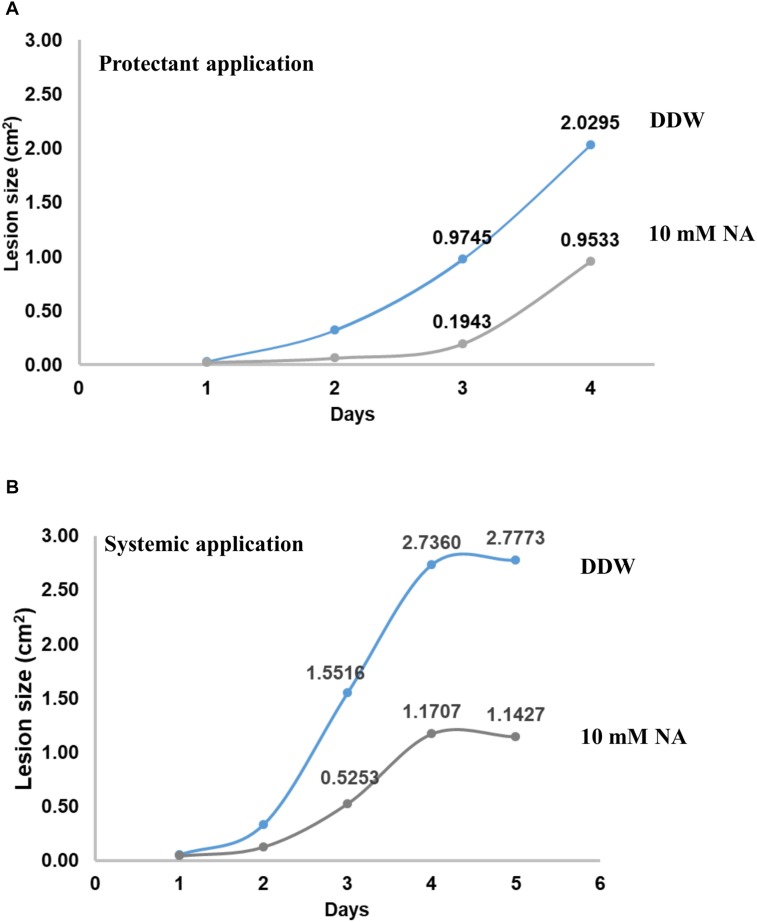
NA suppresses disease symptoms caused by *B. cinerea* in disconnected tomato leaves. **(A)** Leaves from tomato plants were inoculated with 5000 conidia of *B. cinerea* with (gray line) or without (blue line) rinsing in 10 mM NA. The leaves were placed on a water agar plates, the plates were then incubated for the indicated time period. The lesion size was measured every day; the average of the lesion size is presented. **(B)** As for **(A)** but this time the leaves were not rinsed in NA, rather leaves from untreated (blue line) or NA treated (gray line) (10 mM) plants (see legend to Figure 11 and text) were inoculated with *B. cinerea*.

To measure the potential of NA as a protectant pesticide, seven leaves from tomato plants were briefly soaked in either DDW or 10 mM NA. The leaves were inoculated with *B. cinerea* conidia and incubated in a moist chamber for several days. The size of the lesions created by *Botrytis* was measured every day. The average lesion size was smaller in NA-treated leaves compared to the control (Figure 12A). After 3 days the NA inhibitory effect was stronger than the NA inhibitory effect 4 days post inoculation. Three days post inoculation the average size of the lesions caused by *Botrytis* was five times larger in the untreated leaves compared to NA-treated ones. Four days post inoculation the average size of the lesions caused by *Botrytis* was double in untreated leaves compared to the NA-treated ones (Figure 12A). Using the same disconnected leaf assay we measured the ability of NA to serve as a systemic pesticide. Seven leaves taken from 10 mM NA treated tomato plants grown for a week in hydroponic setting (Figure 11B) were inoculated by *B. cinerea* conidia. The average lesion size of NA treated leaves was about 30–40% of untreated control (Figure 12B). The size of the leaves of the treated plants was smaller as indicated earlier (Figure 11B). Therefore, an indirect effect of NA applied systematically on spore germination should be considered.

## Discussion

Here we studied the effect of NA, an inhibitor of Pnc1, on the NAD^+^ status of *F. oxysporum* (Figure 1; French et al., 2010a; Smith et al., 2012). Inhibition of nicotinamidases interferes with NAD^+^ biosynthesis, an important cofactor for various cellular processes such as DNA repair, glycolysis, respiration via electron transport chain, and cell signaling (French et al., 2010b; Fang et al., 2017).

By using RNA sequencing we also looked into the response of *F. oxysporum* to NA. NA inhibited fungal development in *F. oxysporum* and other ascomycetes (Figure 2, 11A). At this stage we do not know why conidia are more sensitive to NA than hyphae. It is possible that the overall permeability of hyphae is lower or their ability to detoxify NA is higher.

Measurements of the total amounts of NAD and its redox status in germinating conidia suggested that NA disrupted the NAD^+^ balance in the cell (Figure 8, 9). The major effect is on the NAD^+^/NADH redox balance, but when the only source for NAD^+^ biosynthesis is within the conidia, it is clear that the amounts of total NAD are also reduced (Figure 8A). The latter physiological condition is similar to the physiological state of germinating conidia during penetration of the host. Comparison of the total NAD levels, NAD^+^ and NAD/NADH ratio between exposures of NA, formaldehyde and H_2_O_2_ revealed the unique effect of NA on NAD^+^ status in the cell. Unlike formaldehyde and H_2_O_2_, NA caused a reduction in the NAD^+^ levels, probably through inhibition of Pnc1 (Figure 6, 8). The NAD^+^/NADH ratio of conidia exposed to NA and formaldehyde were both much lower than in conidia treated with H_2_O_2_; this could be accounted to aldehyde detoxification by aldehyde dehydrogenases. Our data suggest that NA has a combined effect on the NAD^+^ status in the cell, reduction in the amounts through inhibition of the salvage biosynthesis pathway and increase in NADH as a byproduct of aldehyde dehydrogenases. We provide the first support that NA can inhibit nicotinamidase activity in extracts of filamentous fungi (Figure 1, 6). However, there is still a need to further characterize the effect of NA on the different metabolites within the NAD biosynthesis pathway.

The RNA sequencing experiment further strengthened the idea that the major effect of NA is interference with NAD^+^ biology. Most of the genes induced by exposure to NA belonged to the dehydrogenase family. Induction of alcohol dehydrogenases, aldehyde dehydrogenases, and cytochrome P450 oxidoreductases was in agreement with the disturbance of redox balance of the cells caused by the inhibition of NAD^+^ biosynthesis or its redox balance (Figure 3B). A classic cellular response to aldehydes would be oxidative burst or accumulation of reactive oxygen species (ROS) (Bai et al., 2003). ROS trigger antioxidant and ROS scavenging systems to protect the cells from free radicals. Free radicals are controlled by an array of antioxidant enzymes like superoxide dismutase, catalase, glutathione-s-transferase, and ascorbate peroxidase. NA did not provoke a classical oxidative stress response since most of these genes were not induced (Table 1). In fact, ROS measurements suggested that NA generated reductive stress (Figure 5). These results were in agreement with the finding that the NAD^+^/NADH ratio was decreased during NA exposures and not increased as in the response to H_2_O_2_.

**FIGURE 13:**
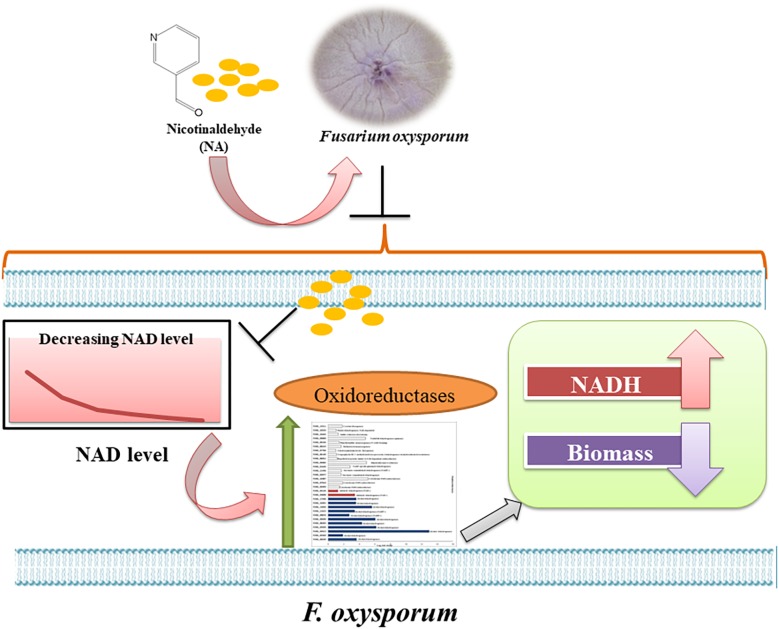
Model of the response of *F. oxysporum* to NA. Summary of the response of *F. oxysporum* to NA based on changes in gene expression, NAD status and ROS levels.

An interesting finding of our study was the inverse correlation of NADH levels and total biomass (total protein) (Figure 9). We found that application of NA led to an increase in the levels of NADH and a subsequent decrease in total protein concentration. This result was consistent both for DDW and PDB grown cultures. The results obtained for formaldehyde also support this correlation. Even though NAD^+^ did not change significantly, NADH levels were significantly increased followed by decrease in the protein concentration (Figure 8E, 9C). In *Aspergillus niger*, decreasing the levels of NADH by inactivating NADH dehydrogenase led to enhanced growth rate and higher protein content (Voulgaris et al., 2012), thus high levels of NADH may be the reason for inhibition of growth caused by NA. Changes in the levels of NAD^+^ and NADH have deleterious effects on the cell. Their importance not only stems from being a cofactor in various enzymatic reactions, but also their deciding role in the fate of various metabolic processes. Depletion of NAD^+^ levels and enhancement of NADH can cause the electron transport chain to be channeled into other pathways rather than being an ATP generating pathway (Watson et al., 2009; Hou et al., 2010; Titov et al., 2016). Less availability of ATP for various cellular processes can lead to growth inhibition. This might be the reason why there was a combined effect between low doses of NA and boscalid (Figure 10). As mentioned above, following NA exposure, oxidoreductases were significantly upregulated, amongst alcohol dehydrogenases were highlighted. Alcohol dehydrogenases convert alcohol to aldehydes, which are further converted to useful acids, and NAD^+^ is reduced in the process. However, in yeast it has been reported that alcohol dehydrogenases can also catalyze the reverse reaction as part of fermentation and to supply NAD^+^ (Thomson et al., 2005). Taken together, we propose that aldehyde dehydrogenases effectively neutralize the toxic effect of the aldehyde group of NA without imposing severe oxidative stress. However, these enzymes use NAD^+^, which during the reaction is converted to NADH. Ultimately, what the fungi sense is the increase in the NADH concentration; the response is activation of alcohol dehydrogenases (summarized in Figure 13).

## Conclusion

In the future, NA or similar compounds combined with other chemicals may be the basis for development of a novel approach to overcome fungal diseases or to enhance the effectiveness of succinate dehydrogenase inhibitors (see differential effect of NA on fungi and plants in Figure 11 and the ability of NA to suppress disease symptoms in disconnected leaves Figure 12). However, based on our results, NA as is cannot serve as a pesticide. Its ability to adhere to leaves or roots should be further investigated and probably enhanced. NA derivatives that affect fungi more specifically are also required as shown in Figure 11. The focus here is basic research. To the best of our knowledge, we performed the first investigation into the question of how fungal plant pathogens respond to chemical interruption of NAD^+^ biology. We also provide the first insight into how fungal plant pathogens respond to aldehydes. A major trend now in plant pathology research is to reveal biocontrol agents that can replace pesticides. The fungistatic or fungicidal activity found in the biocontrol agents is often the result of aldehydes and ketones produced by microorganisms.

## Author Contributions

GA, DW, and NV did the experiments and analyzed the data. JM, LJM, and SC analyzed the data. GA and SC wrote the manuscript.

## Conflict of Interest Statement

The authors declare that the research was conducted in the absence of any commercial or financial relationships that could be construed as a potential conflict of interest.

## Supplementary Material

FIGURE S1 The differential effect of NA on conidial germination and mycelium growth does not stem from the type of media used. **(A)** Agar plugs covered with mycelia of *F. oxysporum* were incubated in PDB or PDB containing 10 mM NA for 5 days shaking at 28°C. Next, the mycelia were collected and put on a clean petri dish. The petri dish was scanned and the surface of the mycelia was calculated using ImageJ software. The surface size of the treated mycelium was 77% of the untreated one. **(B)** 5000–1000 conidia were spotted on PDA plates with 0, 5, and 10 mM NA. The plates were incubated for 5 days at 28°C and scanned.

TABLE S1 RNA sequencing results of *F. oxysporum* exposed to NA conidia were grown in PDB for 5 h followed by 3 h of NA exposure. RNA purification, sequencing, and analysis are described in “Materials and Methods.” Raw and adjusted reads for each gene of four biological repeats, with or without NA exposure, are presented, including fold-increase and significance of the increase.

TABLE S2 List of upregulated and downregulated genes in *F. oxysporum* following NA exposure. Only genes that are either upregulated or downregulated by twofold or more are described. *p*-value threshold for significance was set to be 0.05.

TABLE S3 List of upregulated genes used for STRING analysis. Details in the text.
