# Supplementary figures and images for: How Does Fusarium oxysporum Sense and Respond to Nicotinaldehyde, an Inhibitor of the NAD+ Salvage Biosynthesis Pathway?
Source: Front Microbiol. 2019 Feb 27;10:329. doi: 10.3389/fmicb.2019.00329 (PMC6400851; doi:10.3389/fmicb.2019.00329)

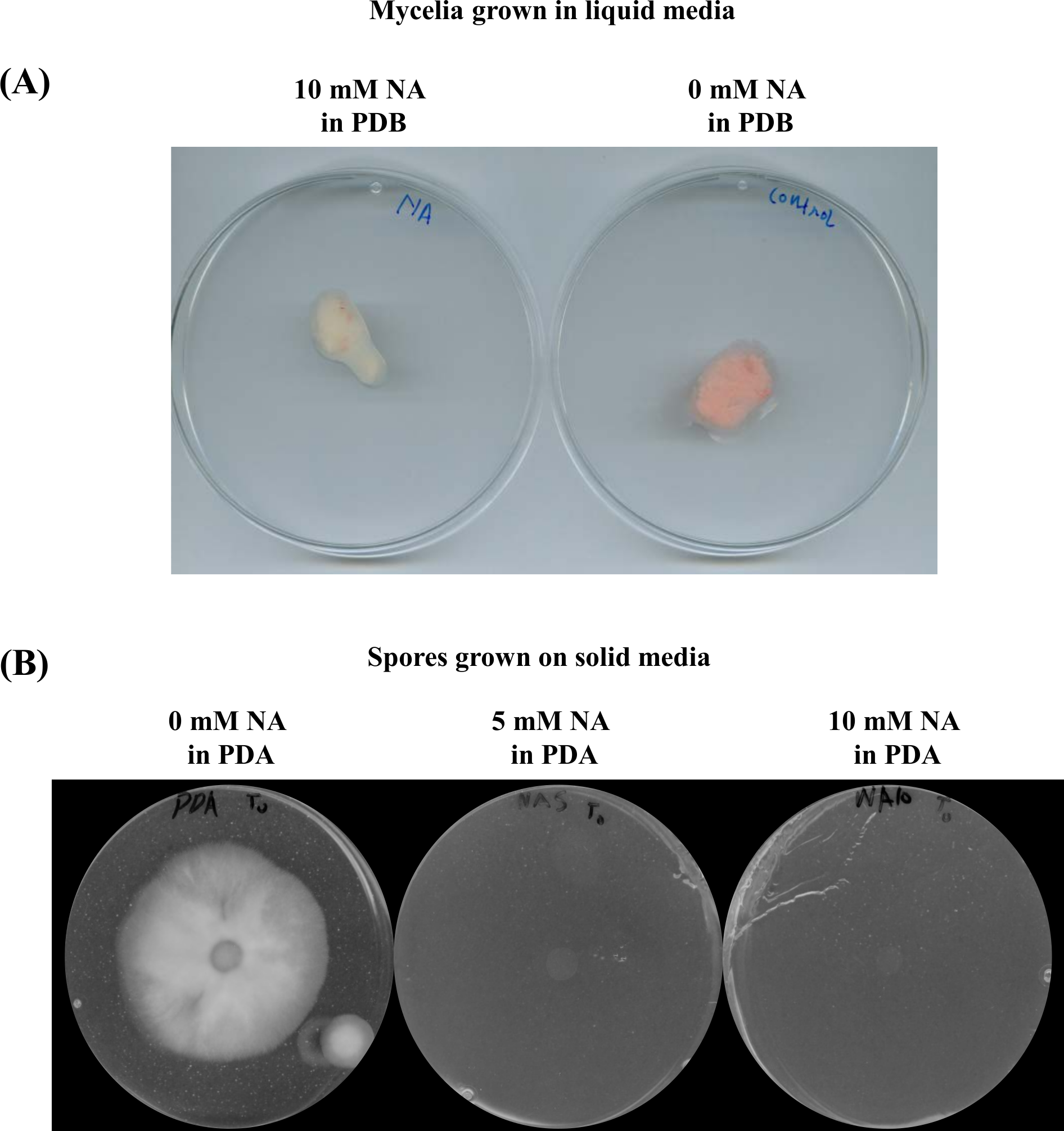

Supplement: FIGURE S1 — The differential effect of NA on conidial germination and mycelium growth does not stem from the type of media used. (A) Agar plugs covered with mycelia of F. oxysporum were incubated in PDB or PDB containing 10 mM NA for 5 days shaking at 28°C. Next, the mycelia were collected and put on a clean petri dish. The petri dish was scanned and the surface of the mycelia was calculated using ImageJ software. The surface size of the treated mycelium was 77% of the untreated one. (B) 5000–1000 conidia were spotted on PDA plates with 0, 5, and 10 mM NA. The plates were incubated for 5 days at 28°C and scanned. [file Image_1.TIF]
